# Gastroprotective Effects of a Polyherbal Formulation (HF33) in Experimental Gastric Ulcer Models and Its Acute Oral Toxicity Evaluation

**DOI:** 10.3390/ph19101565

**Published:** 2026-10-01

**Authors:** Natthakarn Chiranthanut, Sunee Chansakaow, Phraepakaporn Kunnaja, Nirush Lertprasertsuke, Ratchadawan Puangpradab, Nut Koonrungsesomboon

**Affiliations:** 1 Department of Pharmacology, Faculty of Medicine, Chiang Mai University, Chiang Mai 50200, Thailand; 2 Department of Pharmaceutical Sciences, Faculty of Pharmacy, Chiang Mai University, Chiang Mai 50200, Thailand; chsunee@gmail.com; 3 Division of Clinical Chemistry, Department of Medical Technology, Faculty of Associated Medical Sciences, Chiang Mai University, Chiang Mai 50200, Thailand; phraepakaporn.k@cmu.ac.th; 4 Department of Pathology, Faculty of Medicine, Chiang Mai University, Chiang Mai 50200, Thailand; nlertpra@hotmail.com; 5 Queen Sirikit Botanic Garden, The Botanical Garden Organization, Chiang Mai 50180, Thailand; ratchadawanp@hotmail.com

**Keywords:** HF33, polyherbal formulation, gastric ulcer, gastroprotective activity, oxidative stress, acute oral toxicity

## Abstract

**Background/Objectives**: HF33 is a polyherbal formulation developed from Thai folk medicinal knowledge for the management of dyspepsia and gastric ulcer-like symptoms. Its gastroprotective activity and underlying mechanisms have not been systematically investigated. This study aimed to characterize the HF33 formulation and evaluate the gastroprotective activity, possible mechanisms of action, and acute oral toxicity of its ethanolic extract. **Methods**: HF33 formulation powder was characterized according to the Thai Herbal Pharmacopoeia using pharmacognostic, physicochemical, and HPLC-PDA analyses, and the extract was chemically fingerprinted by compact mass spectrometry. Antioxidant activity and total phenolic and flavonoid contents were determined. Gastroprotective activity of the extract (150, 300, and 600 mg/kg, p.o.) was evaluated in male Sprague–Dawley rats using restraint water immersion stress-, indomethacin-, and acidified ethanol (EtOH/HCl)-induced gastric ulcer models. Histopathology, MDA and SOD, gastric mucus content, and gastric secretion parameters were assessed. Acute oral toxicity was evaluated in female Sprague–Dawley rats according to OECD Test Guideline 420. **Results**: Quality characterization established the identity and quality attributes of the HF33 formulation and its extract prior to biological evaluation. The HF33 extract exhibited in vitro antioxidant activity and significantly reduced gastric lesion formation in all three ulcer models. Gastroprotection was associated with preservation of gastric mucosal architecture, increased gastric mucus content, reduced MDA levels, and restored SOD activity, without significant alteration of gastric secretion parameters. No treatment-related toxicity was observed following a single oral administration of 2000 mg/kg. **Conclusions**: The HF33 ethanolic extract exhibited gastroprotective activity across multiple experimental gastric ulcer models. The protective activity was associated with preservation of mucosal integrity, enhancement of the mucus barrier, and attenuation of oxidative damage without affecting gastric acid secretion. These findings support further preclinical investigation of HF33 as a quality-characterized polyherbal formulation with potential gastroprotective properties.

## 1. Introduction

Gastric ulcer continues to be a prevalent gastrointestinal disorder worldwide. It results from an imbalance between aggressive factors, including gastric acid, alcohol exposure, reactive oxygen species (ROS), psychological or physiological stress, and non-steroidal anti-inflammatory drugs (NSAIDs), and endogenous protective mechanisms that maintain gastric mucosal integrity [1,2]. Although acid-suppressive and mucosal-protective agents, including proton pump inhibitors, histamine H2 receptor antagonists, and prostaglandin analogues, are effective in the treatment of gastric ulcer, their long-term use may be associated with adverse effects and ulcer recurrence after treatment discontinuation [3,4]. In addition, gastric ulcer development involves multiple pathological processes beyond excessive acid secretion, including oxidative stress, impaired mucus production, inflammation, reduced mucosal defense, and disruption of epithelial integrity [5]. Therefore, agents capable of enhancing mucosal defense through multiple complementary mechanisms remain of considerable pharmacological interest [6,7].

Natural products have long been explored as potential sources of therapeutic agents for gastrointestinal disorders [2,7]. Among them, polyherbal formulations have attracted particular attention because they contain diverse phytochemical constituents that may produce complementary therapeutic effects, such as antioxidant, anti-inflammatory, and cytoprotective actions [8,9]. However, because these formulations are chemically complex, pharmacological evaluation should be supported by appropriate quality characterization of the tested materials to establish quality attributes and facilitate interpretation of biological findings.

HF33 (herbal formulation No. 33) is a polyherbal formulation developed from Thai folk medicinal knowledge for the management of dyspepsia and gastric ulcer-like symptoms. The formulation includes *Terminalia arjuna* (Roxb. ex DC.) Wight & Arn., *Terminalia chebula* Retz., *Phyllanthus emblica* L., *Caesalpinia sappan* L., and *Maclura cochinchinensis* (Lour.) Corner. Although the individual plant materials included in the formulation have been reported to possess antioxidant and anti-inflammatory properties [10,11,12,13,14,15], the pharmacological effects of the combined formulation have not been systematically investigated. For herbal formulations, quality characterization of the starting material is an important step before biological evaluation because it provides information on the identity and quality of the tested preparation [16]. Pharmacognostic examination, physicochemical analysis, and chemical characterization help establish the quality attributes of the formulation, while characterization of the prepared extract provides complementary information on the test material used for pharmacological studies [17].

The present study aimed to characterize the HF33 formulation and evaluate the gastroprotective effects of its ethanolic extract in experimental gastric ulcer models induced by restraint water immersion stress, indomethacin, and acidified ethanol (EtOH/HCl). These complementary models were selected to represent distinct and clinically relevant factors involved in gastric mucosal injury, including psychological or physiological stress, NSAID exposure, and alcohol-associated mucosal damage. The use of multiple experimental models therefore enabled evaluation of the gastroprotective activity of HF33 across different mechanisms of gastric injury. The HF33 formulation powder was characterized according to the Thai Herbal Pharmacopoeia, while the prepared ethanolic extract was further evaluated by chemical fingerprinting, phytochemical analysis, antioxidant assays, pharmacological studies, mechanistic investigations, and acute oral toxicity assessment.

## 2. Results

### 2.1. Quality Characterization of HF33 Formulation Powder

The quality characteristics of the HF33 formulation powder were evaluated based on pharmacognostic and physicochemical parameters according to the Thai Herbal Pharmacopoeia 2018 [18].

Microscopic examination revealed characteristic botanical structures derived from the constituent herbal materials of the formulation (Supplementary Figure S1). The observed diagnostic features included cork cells, epidermal fragments with stomata, various types of parenchyma, fibers, vessels, starch grains, oil droplets, calcium oxalate crystals, aleurone grains, and other characteristic structures. These findings supported the presence of characteristic botanical structures derived from the herbal materials contained in the HF33 formulation powder.

The physicochemical parameters of the HF33 formulation powder, including loss on drying, total ash, acid-insoluble ash, ethanol-soluble extractive value, and water-soluble extractive value, are presented in Supplementary Table S1. These parameters provided baseline quality characteristics of the starting material used for preparation of the HF33 ethanolic extract.

### 2.2. HPLC-PDA Analysis of Chemical Markers in HF33 Formulation Powder

**Figure 1:**
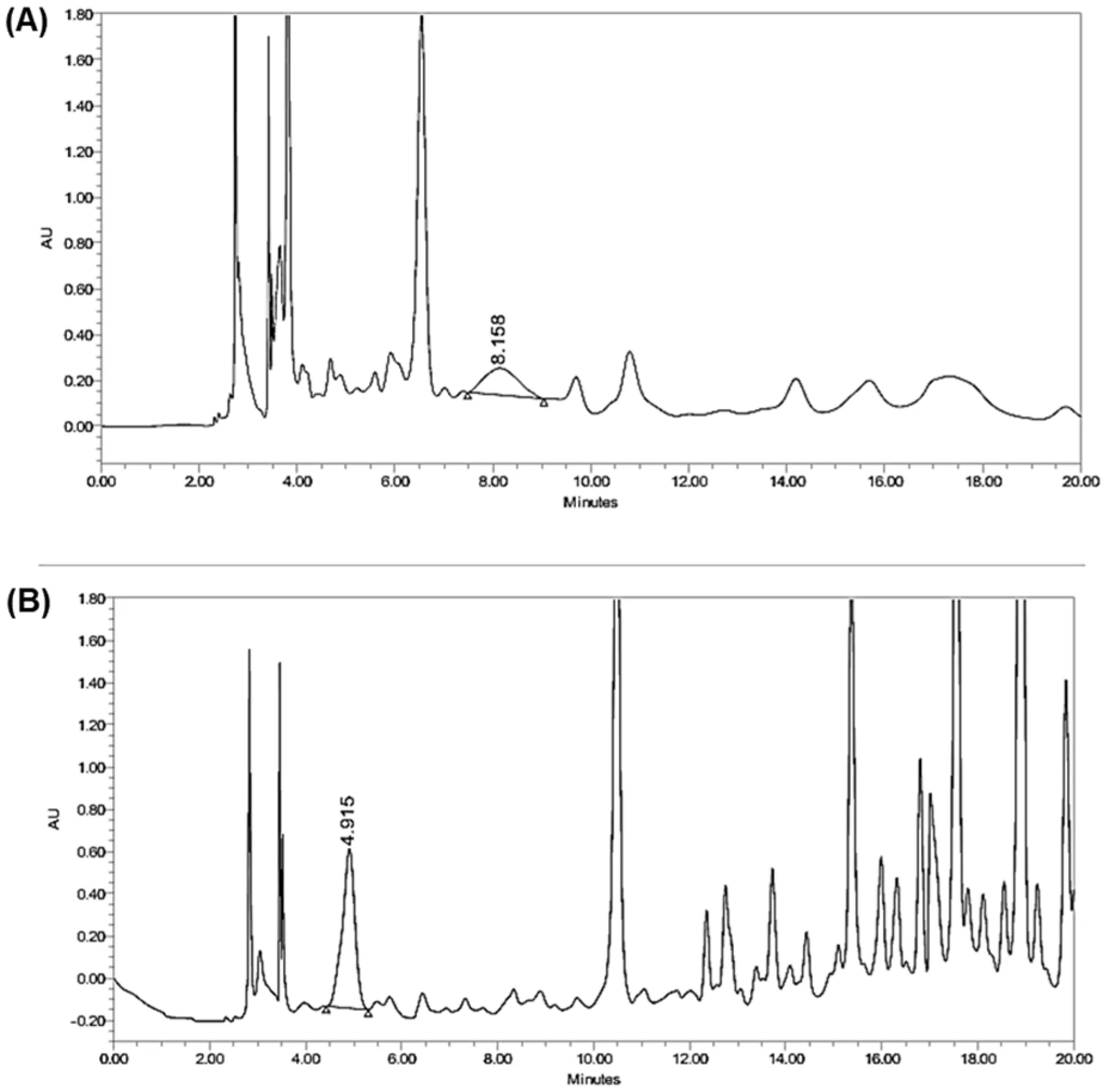
HPLC-PDA chromatograms of HF33 formulation powder. Representative chromatograms showing detection and quantitative analysis of (**A**) tannic acid and (**B**) gallic acid as chemical markers for characterization of the HF33 formulation powder.

Selected chemical markers in the HF33 formulation powder were characterized using high-performance liquid chromatography with photodiode array detection (HPLC-PDA). Gallic acid and tannic acid were identified based on their chromatographic profiles and retention times compared with reference standards (Figure 1). The contents of tannic acid and gallic acid in the HF33 formulation powder were 2.03 ± 0.01 and 3.43 ± 0.01 mg/100 g powder, respectively. Although these compounds are not unique markers of the formulation, their detection provided characteristic chemical information for quality assessment of the HF33 formulation powder.

### 2.3. Preparation and Chemical Characterization of HF33 Ethanolic Extract

**Figure 2:**
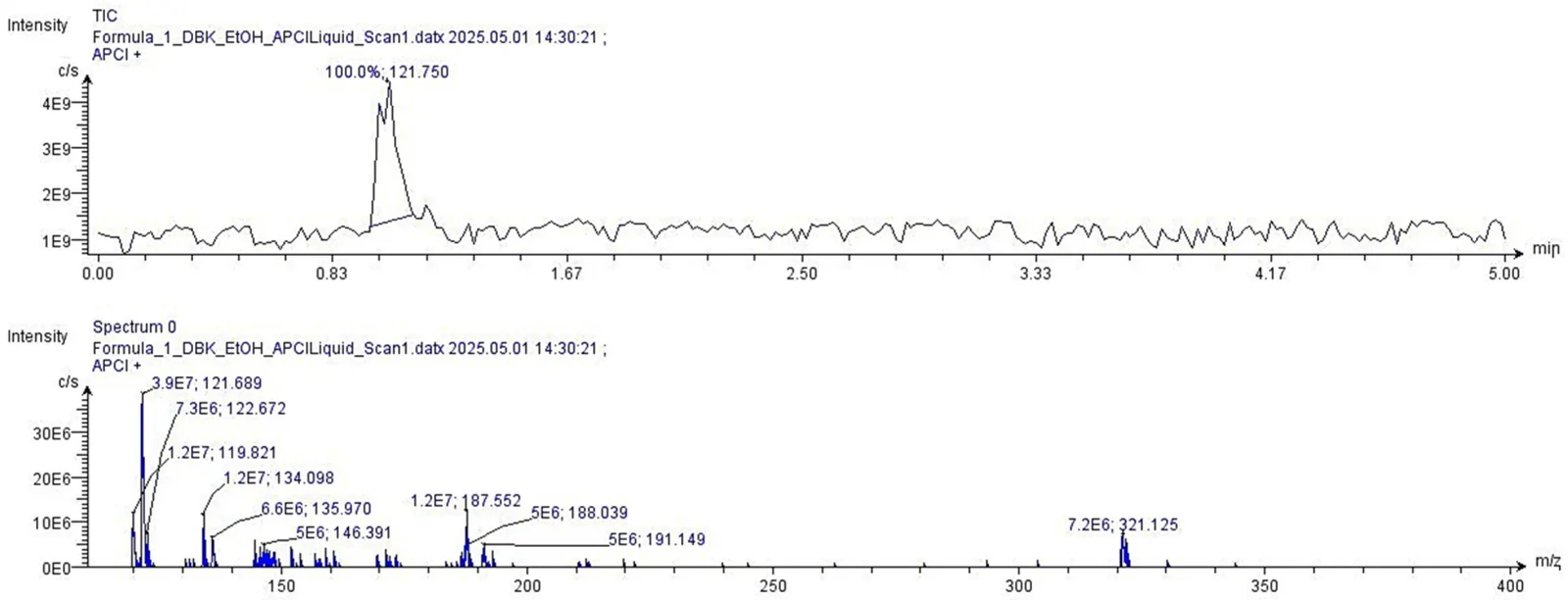
Representative APCI mass spectrum of HF33 ethanolic extract acquired in positive-ion mode (*m*/*z* 100–1200).

Extraction of the HF33 formulation powder using 95% ethanol produced a brownish-yellow extract with an extraction yield of 26.56% (*w*/*w*). The obtained HF33 ethanolic extract was further characterized by compact mass spectrometry (CMS). The APCI mass spectrum of the HF33 ethanolic extract demonstrated a complex chemical profile with multiple molecular ions detected across the analyzed mass range (Figure 2). Several prominent signals, including ions at *m*/*z* 121.689, 134.098, 187.552, and 321.125, among others, were observed (Table 1). The diverse mass spectral pattern reflected the complex chemical composition of the HF33 ethanolic extract, which is consistent with the characteristics of a polyherbal preparation.

### 2.4. Phytochemical Contents and Antioxidant Activity of HF33 Ethanolic Extract

The phytochemical characteristics of the HF33 ethanolic extract were characterized based on its phenolic and flavonoid contents, together with its in vitro antioxidant properties. The extract contained abundant phenolic compounds together with measurable amounts of flavonoids. Among the radical-scavenging assays, the ABTS assay yielded a lower IC_50_ value than the DPPH assay for the HF33 ethanolic extract. In addition, the extract exhibited ferric reducing capacity in the FRAP assay. Detailed quantitative results are presented in Table 2.

### 2.5. Acute Oral Toxicity Profile of HF33 Ethanolic Extract

The acute oral toxicity of the HF33 ethanolic extract was evaluated in female rats according to OECD Guideline 420 [19]. A single oral administration of the HF33 ethanolic extract at 2000 mg/kg caused no mortality or treatment-related clinical signs during the 14-day observation period.

**Figure 3:**
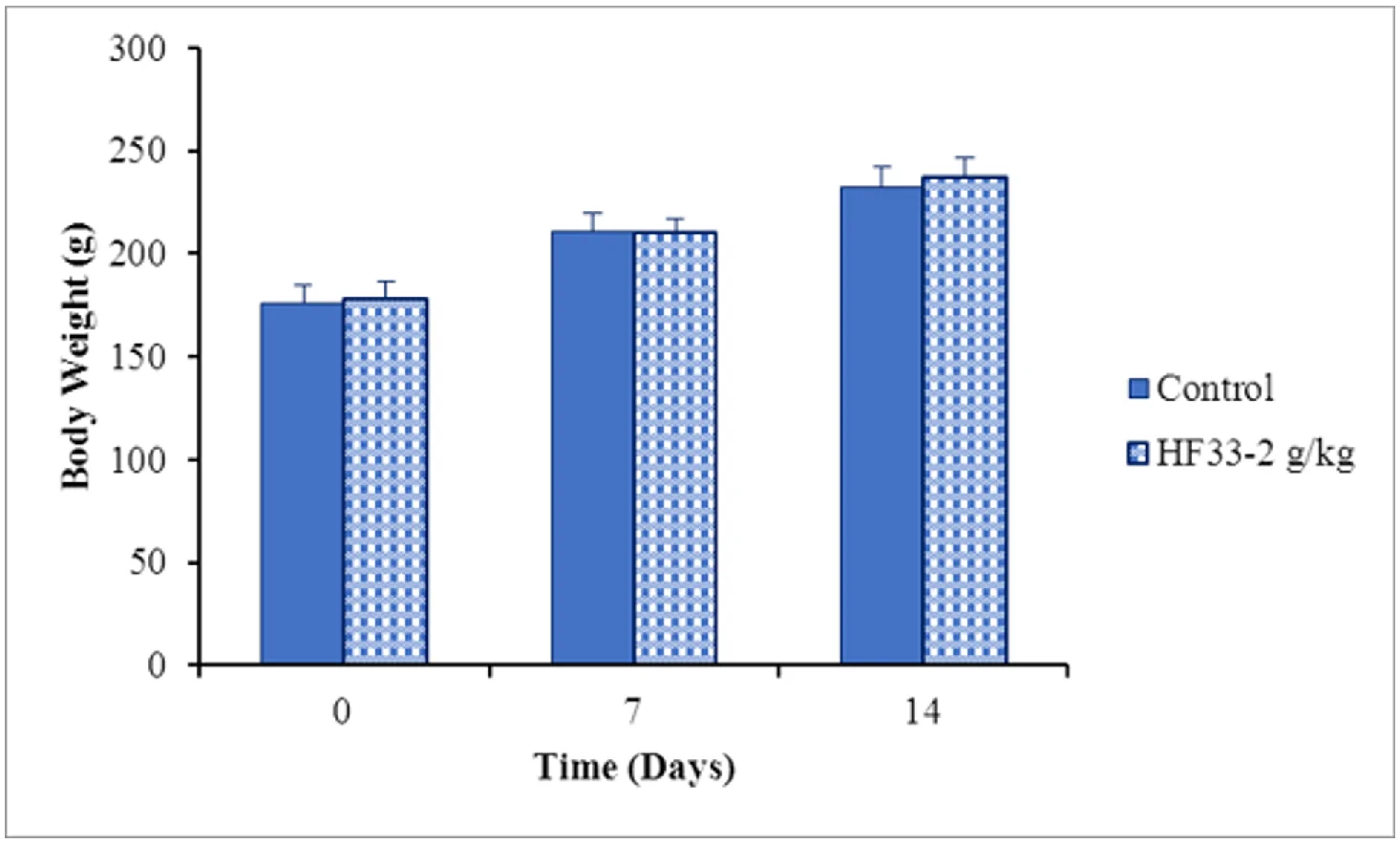
Body weight changes during the acute oral toxicity study of HF33 ethanolic extract in female rats. Data are presented as mean ± SD (*n* = 5). No significant differences were observed between groups.

**Figure 4:**
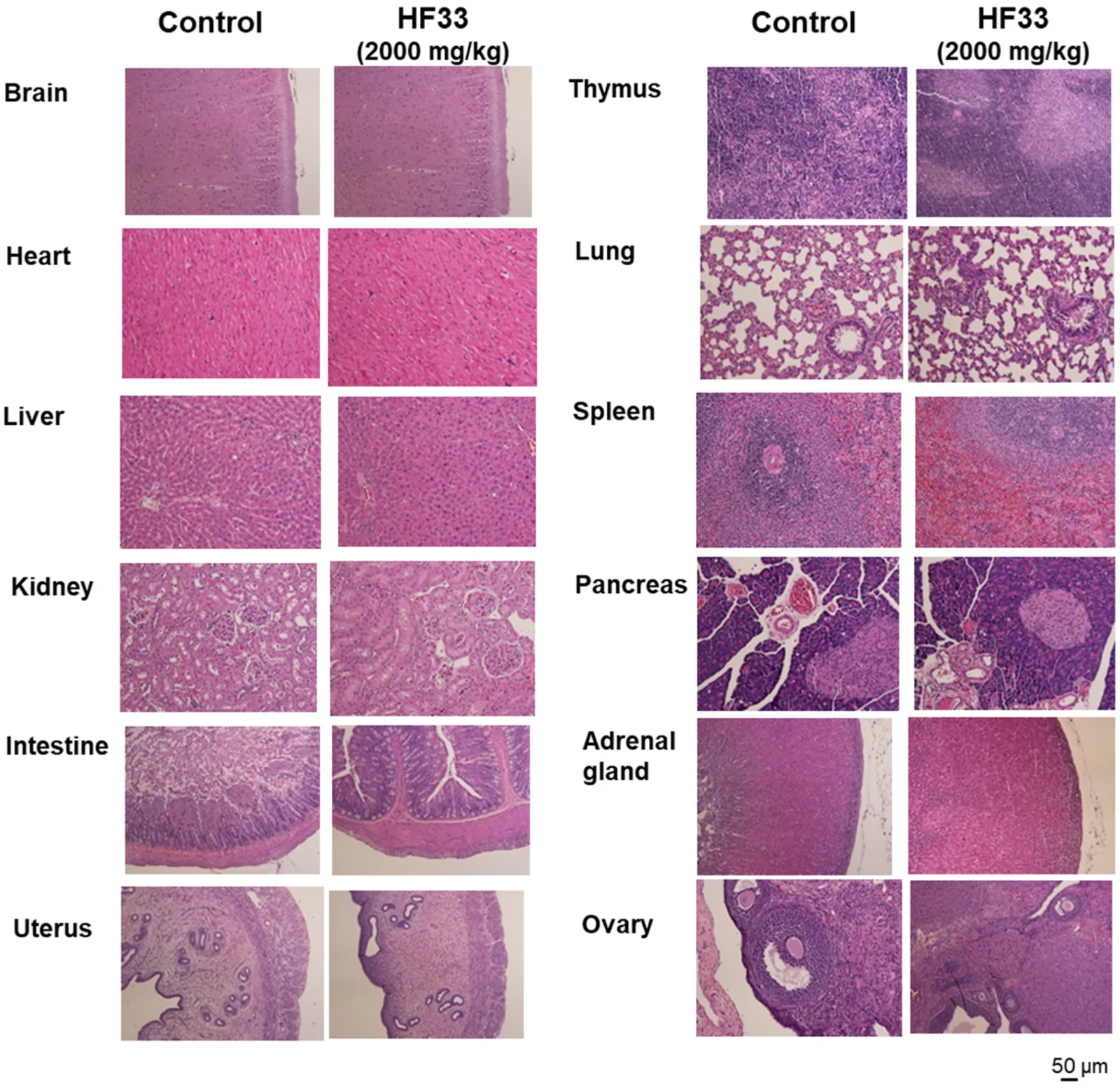
Representative histopathological sections of major organs from female rats following acute oral administration of HF33 ethanolic extract (2000 mg/kg). No treatment-related pathological abnormalities were observed. Original magnification, ×20.

The body weight changes of the HF33-treated rats were comparable with those of the control group throughout the study period (Figure 3). Most hematological and serum biochemical parameters did not differ significantly between the HF33-treated and control groups. However, significant differences were observed in hemoglobin, RBC count, MCV, and MCHC (Table 3 and Table 4). In addition, no significant differences in relative organ weights were observed between the HF33-treated and the control groups, and histopathological examination of major organs revealed no treatment-related abnormalities (Table 5 and Figure 4). Importantly, the observed hematological differences were not accompanied by treatment-related clinical signs, organ-weight changes, or histopathological abnormalities. These findings indicate that the HF33 ethanolic extract did not produce observable acute toxicity following a single oral administration at 2000 mg/kg.

### 2.6. Gastroprotective Effects of HF33 Ethanolic Extract in Experimental Gastric Ulcer Models

The gastroprotective activity of the HF33 ethanolic extract was evaluated in three experimental models of gastric ulcer, including restraint water immersion stress-, indomethacin-, and acidified ethanol (EtOH/HCl)-induced gastric injury.

#### 2.6.1. Effect of HF33 Ethanolic Extract on Restraint Water Immersion Stress-Induced Gastric Ulcers

**Figure 5:**
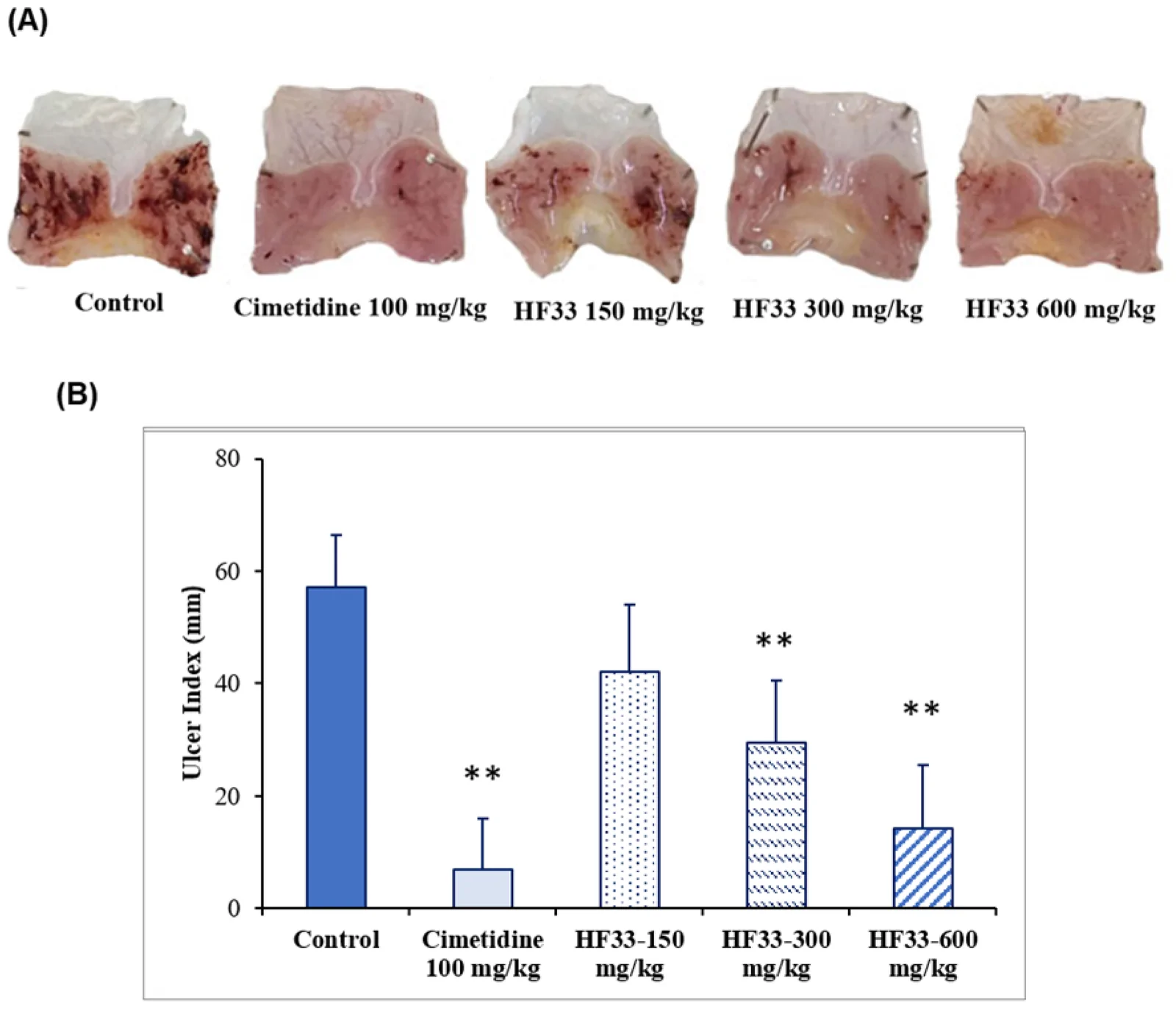
Gastroprotective effects of HF33 ethanolic extract in the restraint water immersion stress-induced gastric ulcer model. (**A**) Representative gastric specimens. (**B**) Ulcer index. Data are presented as mean ± SD (*n* = 6). ** *p* < 0.01 versus ulcer control.

Restraint water immersion stress induced extensive hemorrhagic gastric lesions in the ulcer control group, resulting in a marked increase in the ulcer index (Figure 5A,B). Pretreatment with the HF33 ethanolic extract reduced gastric lesion formation in a dose-dependent manner. Administration of the HF33 ethanolic extract at doses of 150, 300, and 600 mg/kg resulted in ulcer inhibition of 26.31%, 48.28%, and 75.23%, respectively. Linear regression analysis demonstrated a significant inverse linear relationship between HF33 dose and ulcer index (β = −0.833, R^2^ = 0.695, *p* < 0.001), supporting a dose-dependent gastroprotective effect. The reductions in ulcer index the HF33-treated groups were statistically significant at 300 and 600 mg/kg, but not at 150 mg/kg, compared with the ulcer control group. The gastroprotective effect observed at 600 mg/kg was comparable to that of cimetidine, which produced 87.94% inhibition (Figure 5B).

#### 2.6.2. Effect of HF33 Ethanolic Extract on Indomethacin-Induced Gastric Ulcers

**Figure 6:**
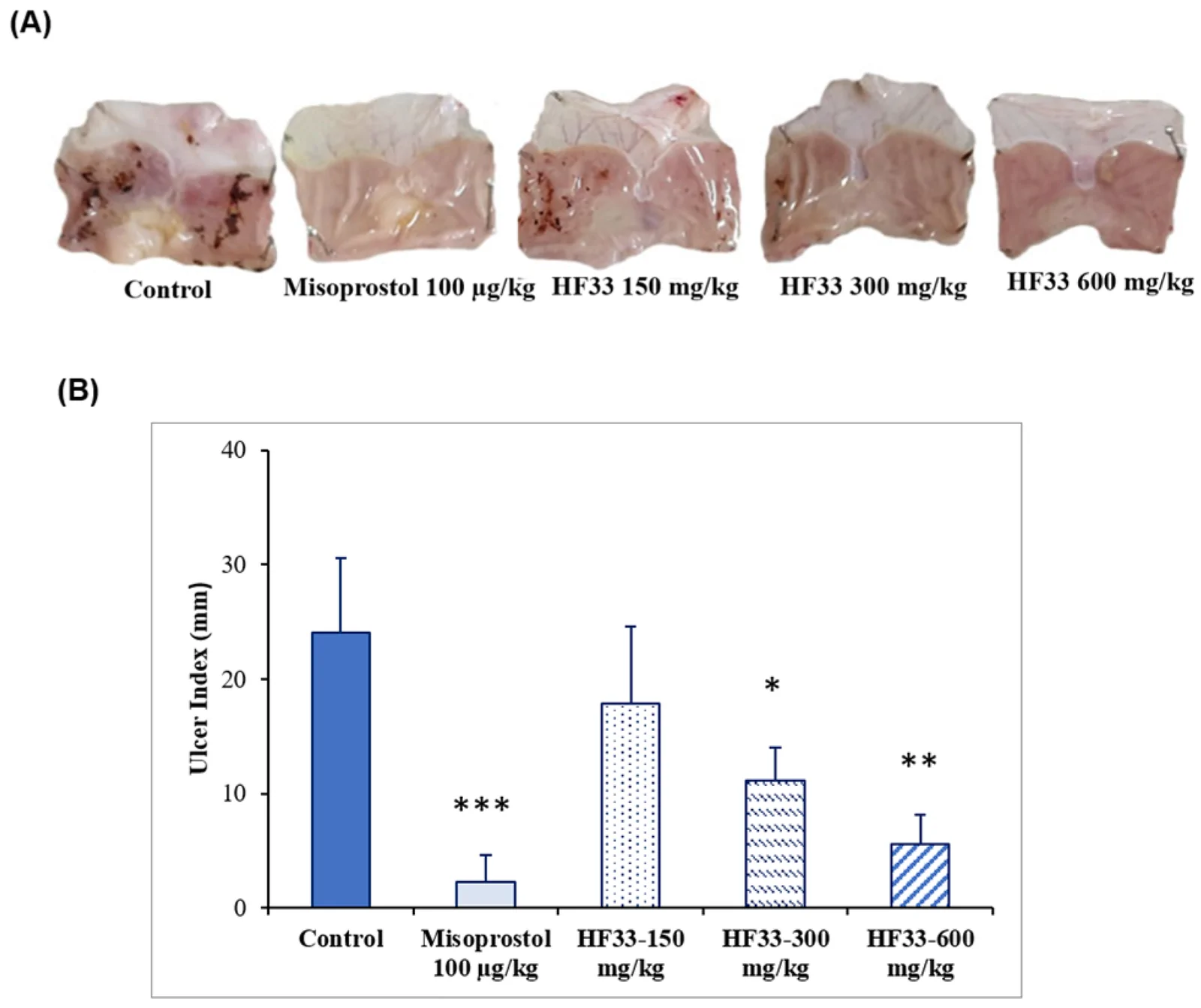
Gastroprotective effects of HF33 ethanolic extract in the indomethacin-induced gastric ulcer model. (**A**) Representative gastric specimens. (**B**) Ulcer index. Data are presented as mean ± SD (*n* = 6). * *p* < 0.05, ** *p* < 0.01 and *** *p* < 0.001 versus ulcer control.

Indomethacin administration produced marked gastric mucosal damage in the ulcer control group, as demonstrated by increased gastric lesions (Figure 6A). Pretreatment with the HF33 ethanolic extract significantly attenuated indomethacin-induced gastric injury at 300 and 600 mg/kg, but not at 150 mg/kg (Figure 6B). The ulcer inhibitory effects of the HF33 ethanolic extract at doses of 150, 300, and 600 mg/kg were 25.73%, 53.74%, and 76.63%, respectively. Nevertheless, linear regression analysis demonstrated a significant inverse linear relationship between HF33 dose and ulcer index (β = −0.811, R^2^ = 0.658, *p* < 0.001), indicating a significant dose–response relationship across the tested doses. The protective effect observed at 600 mg/kg was comparable to that of misoprostol, which produced 90.50% inhibition.

#### 2.6.3. Effect of HF33 Ethanolic Extract on EtOH/HCl-Induced Gastric Ulcers

**Figure 7:**
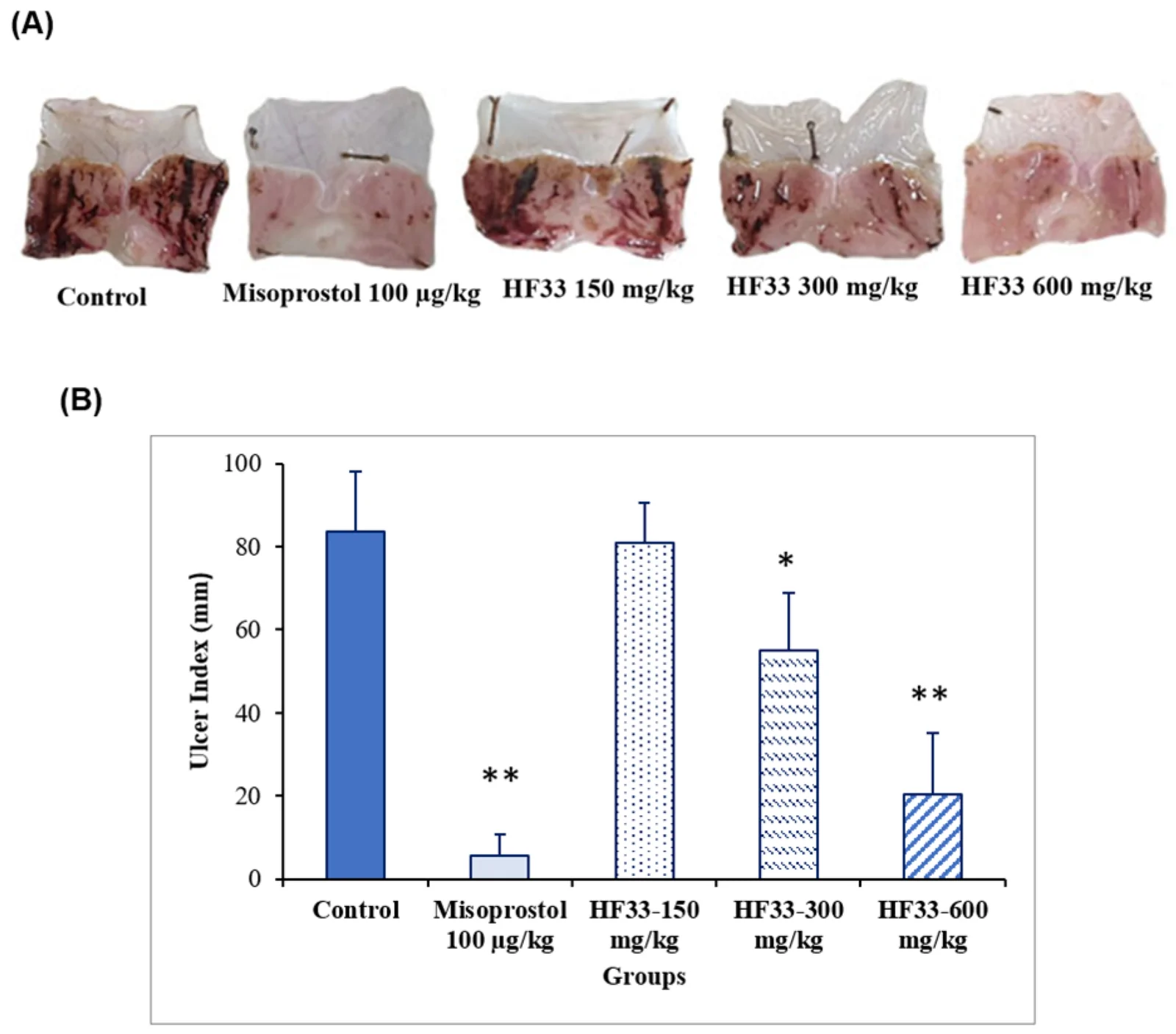
Gastroprotective effects of HF33 ethanolic extract in the EtOH/HCl-induced gastric ulcer model. (**A**) Representative gastric specimens. (**B**) Ulcer index. Data are presented as mean ± SD (*n* = 6). * *p* < 0.05 and ** *p* < 0.01 versus ulcer control.

The EtOH/HCl model produced severe gastric mucosal injury characterized by extensive hemorrhagic lesions in the ulcer control group (Figure 7A). Pretreatment with the HF33 ethanolic extract reduced lesion formation in a dose-dependent manner (Figure 7B). Administration of the HF33 ethanolic extract at doses of 150, 300, and 600 mg/kg resulted in ulcer inhibition of 3.19%, 34.45%, and 75.75%, respectively. Linear regression analysis demonstrated a significant inverse linear relationship between HF33 dose and ulcer index (β = −0.884, R^2^ = 0.781, *p* < 0.001), supporting a dose-dependent gastroprotective effect. The reductions in ulcer index of the extract were statistically significant at 300 and 600 mg/kg, whereas the 150 mg/kg dose did not significantly reduce gastric lesions compared with the ulcer control group. The gastroprotective effect of HF33 ethanolic extract at 600 mg/kg remained lower than that of misoprostol, which produced 93.37% inhibition.

Overall, the HF33 ethanolic extract demonstrated gastroprotective activity in all three experimental gastric ulcer models. The magnitude of protection generally increased with increasing doses, with the highest dose consistently showing the greatest reduction in gastric lesion formation.

### 2.7. Possible Mechanisms Underlying the Gastroprotective Effects of HF33 Ethanolic Extract

To investigate the possible mechanisms underlying the gastroprotective effects of the HF33 ethanolic extract, gastric histopathology, oxidative stress parameters, gastric secretion, and gastric wall mucus content were evaluated using complementary experimental approaches.

#### 2.7.1. Effects of HF33 Ethanolic Extract on Gastric Histopathology and PAS Staining

**Figure 8:**
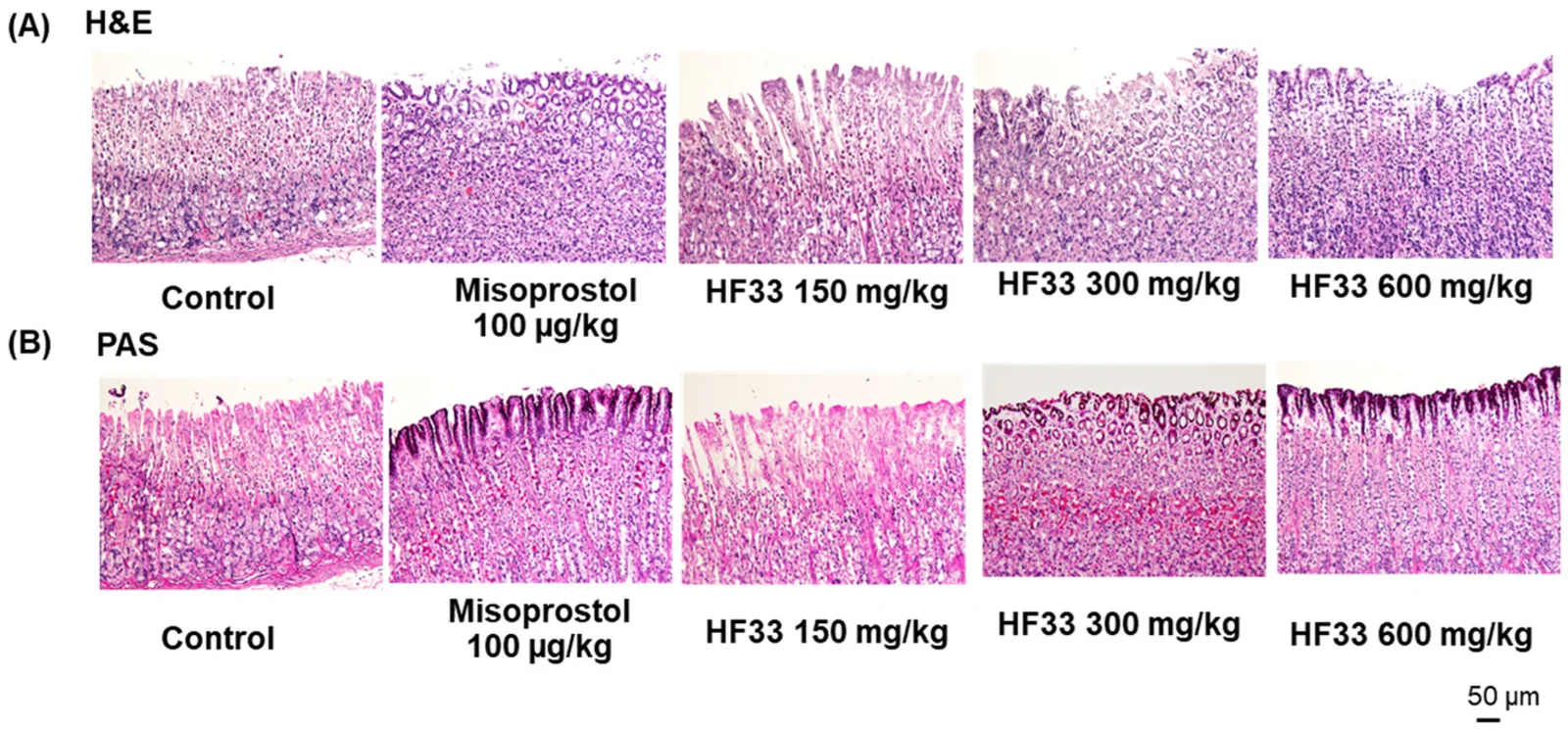
Histopathological evaluation of gastric tissues in the EtOH/HCl-induced gastric ulcer model. Representative gastric tissue sections stained with (**A**) hematoxylin and eosin (H&E) and (**B**) periodic acid-Schiff (PAS). Original magnification, ×20.

Histopathological examination of gastric tissues from the EtOH/HCl-induced gastric ulcer model revealed marked mucosal disruption, epithelial damage, and inflammatory cell infiltration in the ulcer control group compared with the normal group (Figure 8A). Pretreatment with the HF33 ethanolic extract progressively reduced the severity of gastric mucosal injury and improved preservation of gastric tissue architecture with increasing doses. The protective effect of the extract was most evident at 600 mg/kg, which showed improved mucosal morphology compared with the ulcer control group.

Periodic acid–Schiff (PAS) staining was performed to evaluate gastric mucus preservation (Figure 8B). The ulcer control group showed reduced PAS-positive staining following EtOH/HCl administration, indicating disruption of the gastric mucus layer. In contrast, rats pretreated with the HF33 ethanolic extract showed greater PAS-positive staining intensity, particularly at the dose of 600 mg/kg. The PAS staining pattern observed in the HF33 600 mg/kg group was comparable to that of the misoprostol-treated group.

#### 2.7.2. Effects of HF33 Ethanolic Extract on Gastric Oxidative Stress Parameters

**Figure 9:**
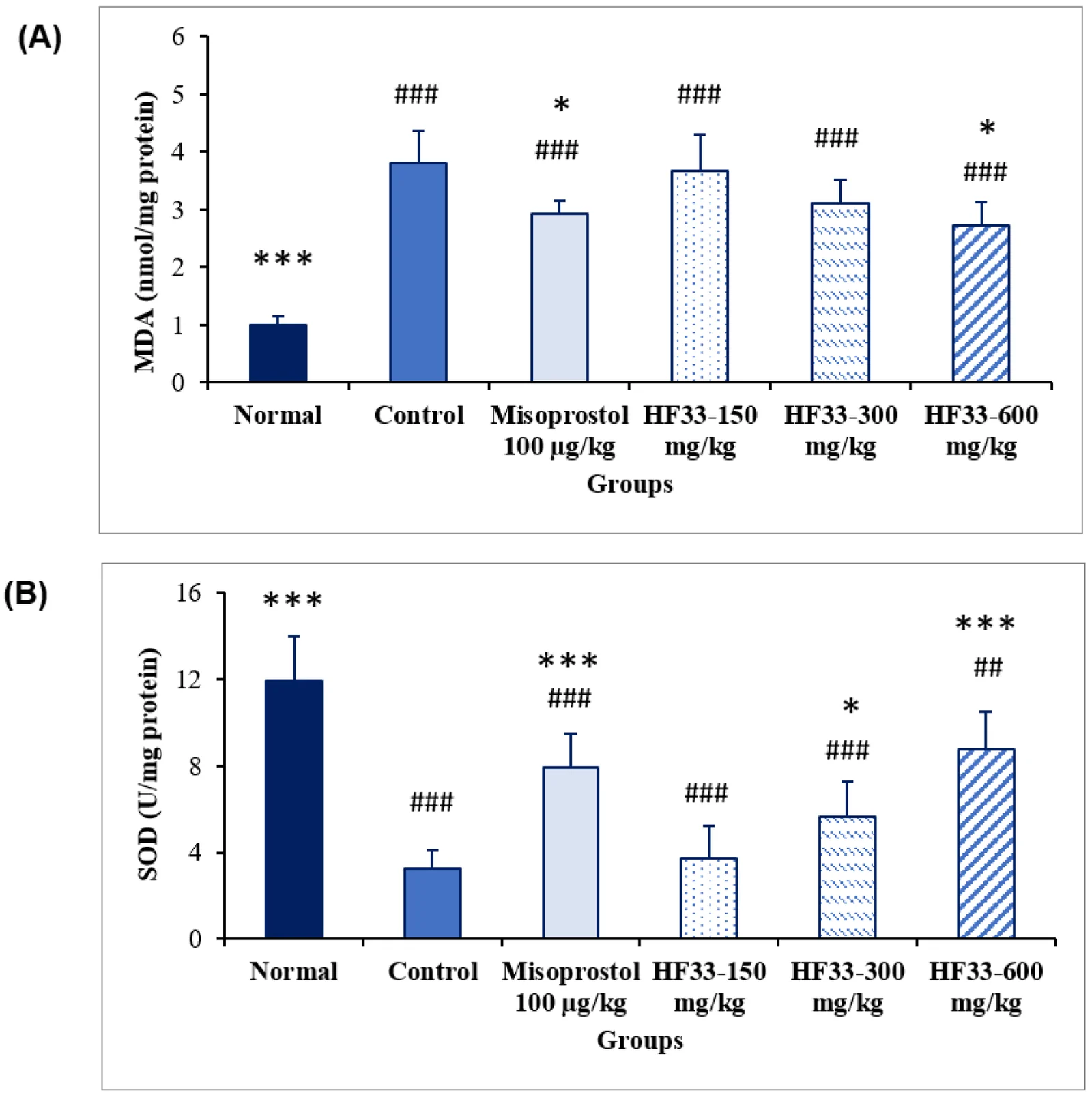
Effects of HF33 ethanolic extract on gastric oxidative stress markers in the EtOH/HCl-induced gastric ulcer model. (**A**) Gastric malondialdehyde (MDA) levels. (**B**) Superoxide dismutase (SOD) activity. Data are presented as mean ± SD (*n* = 6). * *p* < 0.05 and *** *p* < 0.001 versus ulcer control; ## *p* < 0.01 and ### *p* < 0.001 versus normal group.

The effects of the HF33 ethanolic extract on oxidative stress associated with gastric injury were evaluated by measuring gastric malondialdehyde (MDA) levels and superoxide dismutase (SOD) activity (Figure 9A,B). EtOH/HCl administration significantly increased gastric MDA levels and reduced SOD activity compared with the normal group, indicating enhanced lipid peroxidation and impaired antioxidant defense during gastric injury. Pretreatment with the HF33 ethanolic extract significantly reduced MDA levels and increased SOD activity compared with the ulcer control group. Linear regression analysis further demonstrated a significant inverse relationship between HF33 dose and MDA levels (β = −0.658, R^2^ = 0.433, *p* < 0.001), whereas SOD activity showed a significant positive linear relationship with HF33 dose (β = 0.838, R^2^ = 0.703, *p* < 0.001). The effects of the HF33 ethanolic extract on MDA level and SOD activity were more pronounced at the dose of 600 mg/kg.

#### 2.7.3. Effect of HF33 Ethanolic Extract on Gastric Secretion Parameters

**Figure 10:**
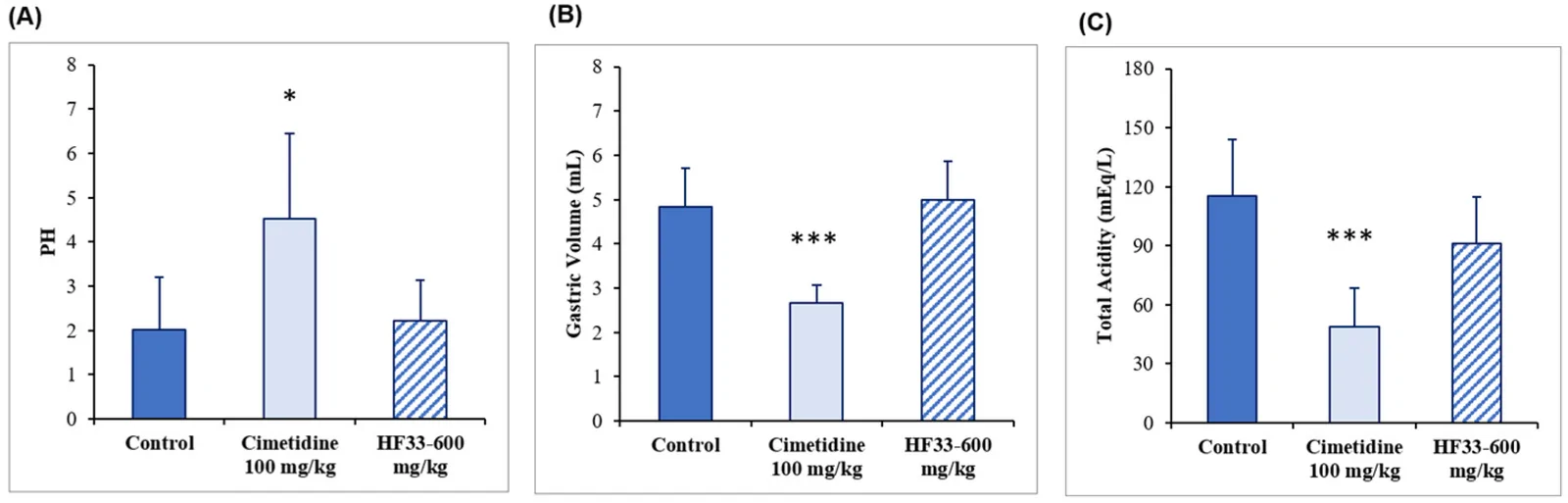
Effects of HF33 ethanolic extract on gastric secretion parameters in pylorus-ligated rats. (**A**) Gastric pH. (**B**) Gastric volume. (**C**) Total acidity. Data are presented as mean ± SD (*n* = 6). * *p* < 0.05 and *** *p* < 0.001 versus control.

To determine whether the gastroprotective activity of the HF33 ethanolic extract was associated with inhibition of gastric acid secretion, gastric secretion parameters were evaluated using the pylorus ligation model (Figure 10). Administration of the HF33 ethanolic extract at 600 mg/kg did not significantly affect gastric volume, gastric pH, or total acidity compared with the control group. In contrast, cimetidine treatment significantly increased gastric pH and reduced total acidity, confirming the responsiveness of the experimental model. These findings indicate that the gastroprotective effects of the HF33 ethanolic extract are unlikely to be mediated primarily through suppression of gastric acid secretion.

#### 2.7.4. Effect of HF33 Ethanolic Extract on Gastric Wall Mucus Content

**Figure 11:**
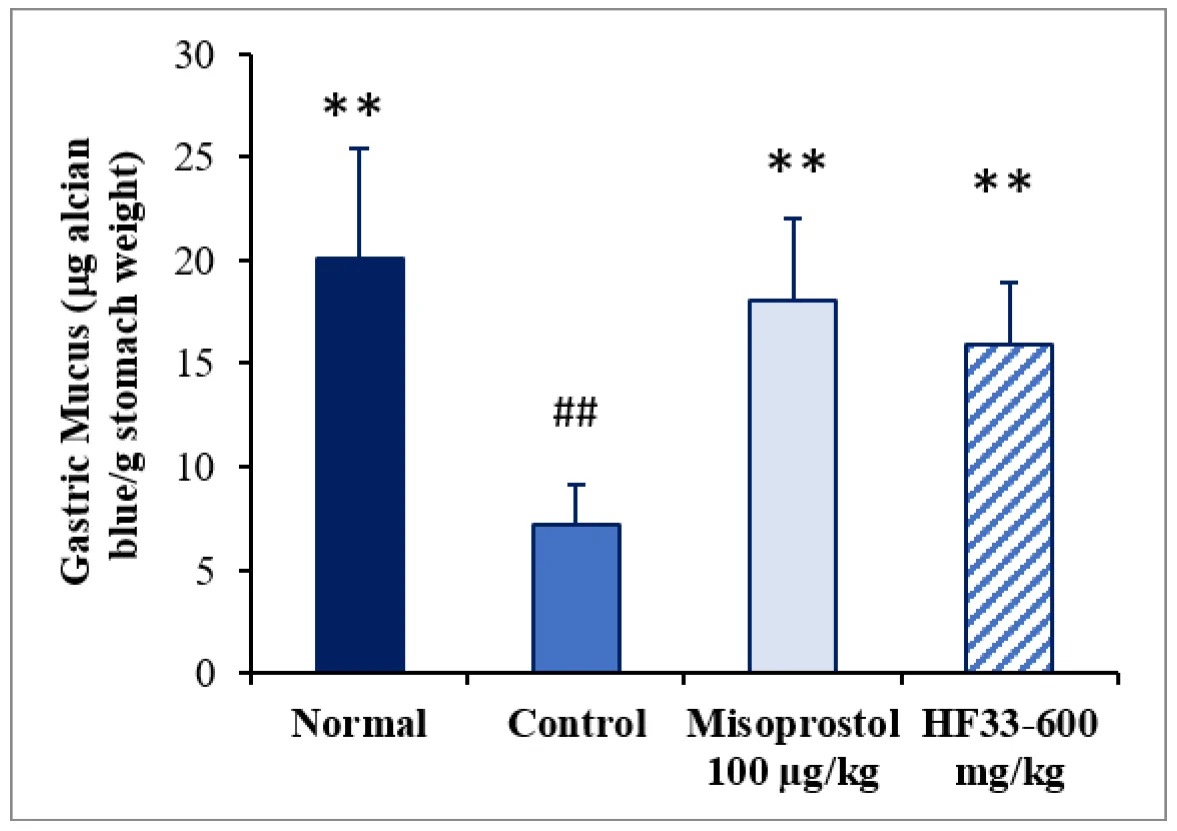
Effects of HF33 ethanolic extract on gastric wall mucus content in rats. Data are presented as mean ± SD (*n* = 6). ** *p* < 0.01 versus ulcer control; ## *p* < 0.01 versus normal group.

Gastric wall mucus content was assessed using the Alcian blue binding assay (Figure 11). EtOH/HCl administration significantly decreased gastric wall mucus content relative to the normal group, indicating impairment of the gastric mucus barrier. Pretreatment with the HF33 ethanolic extract at 600 mg/kg significantly restored gastric wall mucus content relative to the ulcer control group. Although the value observed after HF33 treatment remained numerically below that of the normal group, no significant difference was detected between these groups.

## 3. Discussion

The present study demonstrated that the ethanolic extract of HF33, a quality-characterized polyherbal formulation, exerted significant gastroprotective effects in multiple experimental gastric ulcer models in rats. The HF33 ethanolic extract reduced gastric lesion formation induced by restraint water immersion stress, indomethacin, and acidified ethanol (EtOH/HCl), representing different pathogenic mechanisms of gastric injury. The protective effects were associated with preservation of gastric mucosal integrity, enhancement of the gastric mucus barrier, and attenuation of oxidative stress, while gastric secretion parameters were not significantly altered. In addition, the HF33 ethanolic extract showed no evidence of acute oral toxicity following a single administration at 2000 mg/kg. These findings indicate that HF33 primarily protects the gastric mucosa through enhancement of mucosal defense mechanisms rather than suppression of gastric acid secretion.

Different experimental ulcer models provide complementary information regarding the mechanisms underlying gastric mucosal injury and protection. Restraint water immersion stress induces gastric lesions through a combination of reduced mucosal blood flow, neurohumoral activation, inflammation, and oxidative stress [20,21]. Indomethacin-induced gastric injury is mainly mediated by inhibition of prostaglandin synthesis, resulting in impaired mucosal defense, reduced mucus and bicarbonate secretion, and increased susceptibility to oxidative damage [22,23]. In contrast, EtOH/HCl causes rapid gastric mucosal injury through direct epithelial disruption, depletion of the mucus barrier, oxidative stress, and microvascular damage [24,25]. The ability of the HF33 ethanolic extract to attenuate gastric injury across these complementary models suggests that its protective effects involve multiple components of gastric mucosal defense rather than targeting a single ulcerogenic pathway.

The preservation of gastric mucosal integrity appeared to be an important component of the gastroprotective action of the HF33 ethanolic extract. In the EtOH/HCl-induced gastric ulcer model, the HF33 ethanolic extract reduced epithelial damage and inflammatory changes as demonstrated by histopathological examination. These findings were further supported by PAS staining and quantitative assessment of gastric wall mucus content, which showed improved preservation of the mucus layer in the HF33-treated animals. Gastric mucus represents a primary defensive barrier that protects the gastric epithelium from acid, digestive enzymes, and other damaging agents while supporting epithelial repair processes [26,27]. Therefore, enhancement and preservation of the gastric mucus barrier may contribute substantially to the cytoprotective activity observed following HF33 administration.

Oxidative stress is recognized as a major contributor to gastric mucosal injury in various experimental ulcer models, particularly in EtOH/HCl-induced gastric damage [28,29]. Excessive generation of reactive oxygen species promotes lipid peroxidation, membrane disruption, and impairment of endogenous antioxidant defense systems, leading to further mucosal injury [30,31]. In the present study, the HF33 ethanolic extract significantly reduced gastric malondialdehyde (MDA) levels and restored superoxide dismutase (SOD) activity following EtOH/HCl administration, suggesting attenuation of oxidative damage and improvement of antioxidant defense capacity in gastric tissue [24,32]. These findings were consistent with the in vitro antioxidant activity of the extract demonstrated by DPPH, ABTS, and FRAP assays, supporting a possible contribution of antioxidant mechanisms to the observed gastroprotective effects. However, MDA and SOD were measured in whole gastric tissue homogenates and therefore did not distinguish between the mucosal and submucosal compartments. Moreover, these parameters provide indirect evidence of oxidative stress rather than direct measurement of ROS. Therefore, the present findings should be interpreted as evidence of altered oxidative status rather than definitive evidence of a direct ROS-mediated mechanism. Future studies incorporating complementary ROS detection approaches, additional oxidative stress and antioxidant biomarkers, and spatially resolved analyses could further clarify the role of ROS in the observed gastroprotective effects [33,34].

Phytochemical characterization of the HF33 ethanolic extract provided additional information regarding its chemical composition and potential contributors to its biological activity. The extract exhibited relatively high total phenolic content (TPC) and measurable total flavonoid content (TFC). Previous studies have reported gastroprotective and antioxidant properties for several plant components included in HF33, including *Phyllanthus emblica* [10], *Terminalia arjuna* [11,12], *Terminalia chebula* [13,14], and *Caesalpinia sappan* [35]. Several of these plant materials have also been reported to contain phenolic constituents associated with antioxidant and cytoprotective activities [36,37,38]. Therefore, phenolic constituents may contribute, at least partially, to the antioxidant and gastroprotective effects of the HF33 ethanolic extract. However, this possibility remains hypothetical because the present study did not isolate or individually evaluate the phenolic or flavonoid constituents responsible for the observed effects. TPC and TFC assays provide overall estimates of these compound classes rather than identification or quantification of individual bioactive constituents. Given the complex composition of HF33, the observed biological effects may reflect contributions from multiple phytochemical constituents rather than a single identified compound [9,39].

Quality characterization is an essential consideration in pharmacological studies involving complex herbal formulations because it supports the identity, consistency, and suitability of the tested materials prior to biological evaluation [40,41]. In the present study, the HF33 formulation powder was characterized according to the Thai Herbal Pharmacopoeia 2018 using pharmacognostic and physicochemical parameters [18]. Microscopic examination, extractive values, ash determination, and HPLC-PDA analysis provided complementary information regarding the quality attributes of the formulation. Gallic acid and tannic acid were selected as reference markers because they were detectable in the HF33 formulation and suitable for routine analytical determination. Neither compound was considered a specific active constituent of HF33; rather, the HPLC-PDA analysis was used to support quality control and batch-to-batch standardization of the formulation [42,43].

Further characterization of the HF33 ethanolic extract using compact mass spectrometry (CMS) generated a qualitative chemical fingerprint reflecting the complex and multicomponent nature of the polyherbal preparation. Such chemical fingerprinting approaches are useful for documenting extract characteristics and supporting the quality, identity, and reproducibility of herbal preparations, particularly for polyherbal formulations in which the biological activity may arise from the combined effects of multiple constituents rather than from a single compound [44,45]. The primary purpose of the present chemical characterization was to support the quality and reproducibility of the tested HF33 preparation rather than to comprehensively identify individual constituents or establish their chemical structures. Comprehensive LC-MS/MS analysis could provide more detailed information on the chemical composition of HF33; however, such detailed constituent identification was beyond the scope of the present study. Accordingly, the current chemical characterization should be regarded as supporting the characterization and reproducibility of the tested preparation rather than as evidence identifying individual constituents or the compounds responsible for its gastroprotective activity. Advanced phytochemical profiling, metabolomics, and bioactivity-guided approaches would be required to further elucidate the individual constituents and their potential contributions to the biological effects of HF33.

The role of gastric acid secretion in the protective activity of the HF33 ethanolic extract was investigated using the pylorus ligation model. Inhibition of gastric acid secretion represents a major mechanism of action of several conventional anti-ulcer drugs, particularly proton pump inhibitors and histamine H2-receptor antagonists [46,47]. However, the HF33 ethanolic extract did not significantly alter gastric volume, gastric pH, or total acidity in pylorus-ligated rats. These findings suggest that the gastroprotective activity of HF33 is unlikely to be mediated primarily through suppression of gastric acid secretion. Instead, the observed protection appears to involve enhancement of mucosal defense mechanisms, including preservation of the mucus barrier and reduction of oxidative damage, which are important protective processes against gastric injury [48,49].

In the present study, the HF33 ethanolic extract at the highest dose of 600 mg/kg produced greatest gastroprotective effects in the experimental rat models. The doses of 150, 300, and 600 mg/kg were selected as an experimental dose range based on a previous in vivo study of another polyherbal formulation using the same experimental gastric ulcer models [49]. These doses were used to evaluate the gastroprotective effects of HF33 across different dose levels and were not intended to establish a therapeutic dose for human use. Direct extrapolation of the effective dose from rats to humans is not appropriate without consideration of pharmacokinetic exposure, dose scaling, formulation characteristics, and safety margins. Further dose-optimization, pharmacokinetic, toxicity, and clinical studies are required to determine the appropriate therapeutic dose in humans.

The acute oral toxicity study provided preliminary evidence regarding the safety profile of the HF33 ethanolic extract. Following a single oral administration of 2000 mg/kg, no mortality, treatment-related clinical abnormalities, significant changes in biochemical parameters, alterations in relative organ weights, or histopathological abnormalities were observed. Although four hematological parameters, including hemoglobin, RBC, MCV, and MCHC, were significantly different in the HF33-treated group compared with the control groups, these differences were not considered clinically significant, as all values remained within the normal ranges reported for healthy Sprague Dawley rats [50]. According to OECD Test Guideline 420, the absence of mortality at this dose suggests that the median lethal dose (LD_50_) is expected to exceed 2000 mg/kg under the conditions of this study [19]. These findings provide preliminary evidence of a favorable acute oral safety profile of the HF33 ethanolic extract under the conditions tested. However, further repeated-dose toxicity studies and pharmacokinetic investigations are required to better characterize long-term tolerability and systemic exposure of HF33 under conditions relevant to potential therapeutic use.

Several limitations of the present study should be acknowledged. First, HF33 is a complex polyherbal formulation, and the individual phytochemical constituents responsible for its gastroprotective effects have not yet been identified. Although pharmacognostic and physicochemical characterization, HPLC-PDA marker analysis, and chemical fingerprinting were performed, these approaches primarily support the identity, quality, and reproducibility of the tested materials rather than establish the individual constituents responsible for the observed biological effects [51,52]. In particular, the CMS analysis provided a qualitative chemical fingerprint to support characterization of the tested preparation but was not designed for comprehensive identification or structural confirmation of individual constituents. Future studies using advanced phytochemical profiling, metabolomics, and bioactivity-guided fractionation may facilitate identification of the active constituents responsible for the observed pharmacological effects [53,54]. Second, MDA and SOD were measured in whole gastric tissue homogenates and therefore did not distinguish between the mucosal and submucosal compartments [55]. Moreover, these parameters provide indirect evidence of oxidative stress rather than direct measurement of ROS. Complementary ROS detection approaches, such as dihydroethidium (DHE) fluorescence staining, together with spatially resolved analyses and additional oxidative stress biomarkers, could provide further mechanistic information [33,56]. In addition, inflammatory biomarkers were not evaluated in the present study, although inflammatory responses are an important component of gastric mucosal injury and may contribute to the pathogenesis of gastric ulceration [55,57]. Therefore, whether modulation of inflammatory pathways contributes to the gastroprotective effects of HF33 remains to be established and warrants further investigation. Third, while the present study investigated important protective mechanisms, including gastric mucus preservation and attenuation of oxidative damage, the underlying molecular signaling pathways remain to be elucidated. Finally, the experimental gastric ulcer models used in this study do not fully reproduce the multifactorial pathophysiology of human gastric ulcer disease [58,59]; therefore, the generalisability of these findings to other species and their translation to human gastric disease remain to be established.

Overall, the present study demonstrates that a quality-characterized HF33 ethanolic extract exhibits gastroprotective activity in multiple experimental gastric ulcer models. The protective effects are associated with preservation of gastric mucosal integrity, enhancement of the gastric mucus barrier, and attenuation of oxidative stress rather than inhibition of gastric acid secretion. By integrating appropriate quality characterization with pharmacological evaluation, this study provides preclinical evidence supporting further investigation of HF33 as a polyherbal formulation with potential gastroprotective properties.

## 4. Materials and Methods

### 4.1. Materials and Reagents

The dried plant materials, including *Terminalia arjuna* (Roxb. ex DC.) Wight & Arn., *Terminalia chebula* Retz., *Phyllanthus emblica* L., *Caesalpinia sappan* L., and *Maclura cochinchinensis* (Lour.) Corner, were provided by Twin Lotus Company, Limited (Bangkok, Thailand). Wannaree Charoensup, a botanist at the Faculty of Pharmacy, Chiang Mai University, authenticated the plant materials, which were subsequently deposited at the Medicinal Plant Innovation Center, Faculty of Pharmacy, Chiang Mai University, Chiang Mai, Thailand, under voucher numbers DBK 003–DBK 007, respectively. The materials were then processed according to the manufacturer’s preparation procedure and combined to obtain the HF33 formulation powder.

Reference standards, including gallic acid, tannic acid, and quercetin, were purchased from Sigma-Aldrich (St. Louis, MO, USA). All solvents and reagents used for chromatographic, phytochemical, and biochemical analyses were of analytical or HPLC grade.

### 4.2. Pharmacognostic and Physicochemical Evaluation of HF33 Formulation Powder

The formulation powder was characterized prior to extraction to verify its identity and quality before use in subsequent experiments. The identity and quality characteristics of the HF33 formulation powder were evaluated according to the official procedures described in the Thai Herbal Pharmacopoeia 2018 [18].

#### 4.2.1. Microscopic Characterization

Microscopic examination was performed to identify diagnostic anatomical characteristics of the powdered formulation and to support the botanical authentication of its constituent herbal materials. The powdered sample was mounted on glass slides and examined using a Nikon Eclipse E200 photomicroscope (Jiangnan, Nanjing, China) at 40× magnification, equipped with a Canon EOS 760D digital camera (Canon Inc., Tokyo, Japan). Images were captured using EOS Utility 3 software (Canon Inc., Tokyo, Japan). Characteristic microscopic features, including epidermal structures, fibers, vessels, starch grains, crystals, oil droplets, and other diagnostic structures, were recorded.

#### 4.2.2. Physicochemical Evaluation

Physicochemical parameters of the HF33 formulation powder were determined according to the Thai Herbal Pharmacopoeia 2018, including loss on drying, total ash, acid-insoluble ash, ethanol-soluble extractive value, and water-soluble extractive value. All analyses were performed following the official pharmacopoeial procedures.

#### 4.2.3. High-Performance Liquid Chromatography (HPLC-PDA) Analysis of HF33 Formulation Powder

Chemical marker analysis of the HF33 formulation powder was performed using high-performance liquid chromatography with photodiode array detection (HPLC-PDA). This analysis was performed for quality characterization and chemical standardization of the HF33 formulation powder. It was conducted by the Thailand Institute of Scientific and Technological Research (TISTR), Pathum Thani, Thailand, a national research organization that provides analytical and testing services, including services for herbal products. HPLC analysis was performed using a Waters Alliance 2695 system equipped with a 2998 photodiode array detector and Empower 3 software Build 3471, Feature Release 2 (Waters Corp., Milford, MA, USA). Separation was achieved on a pHedure C18 column (250 × 4.6 mm, 5 μm) maintained at 30 °C, with detection at 280 nm. Gallic acid and tannic acid were selected as chemical markers for characterization of the HF33 formulation powder. For sample preparation, the HF33 formulation powder (1500 mg) was extracted with methanol, sonicated for 15 min, and filtered through a 0.20 μm PTFE membrane before injection. The injection volume was 10 μL. Tannic acid was analyzed under isocratic conditions using 0.1% acetic acid (pH 2.5) and acetonitrile (87:13, *v*/*v*) as the mobile phase at a flow rate of 1.0 mL/min. Gallic acid was analyzed using gradient elution with the same solvent system, with acetonitrile increased from 5% to 40% over 35 min at a flow rate of 1.0 mL/min.

### 4.3. Preparation of HF33 Ethanolic Extract

The HF33 formulation powder was subjected to continuous extraction with 95% ethanol. The extract was filtered and concentrated under reduced pressure using a rotary evaporator (Buchi, Labortechnik AG, Flawil, Switzerland) to obtain a brownish-yellow crude extract. The extraction yield was calculated based on the weight of the dried extract relative to the initial weight of the HF33 formulation powder. The obtained extract was subsequently spray-dried and stored at 4 °C until further analysis and experimental use.

### 4.4. Compact Mass Spectrometry Fingerprinting of HF33 Ethanolic Extract

The chemical fingerprint of the HF33 ethanolic extract was evaluated using compact mass spectrometry (CMS; Advion Inc., Ithaca, NY, USA) equipped with an atmospheric-pressure chemical ionization (APCI) source. Prior to analysis, the HF33 ethanolic extract was dissolved in HPLC-grade methanol at a concentration of 0.01 mg/mL and passed through a 0.22 μm syringe filter (Sigma-Aldrich, Darmstadt, Germany). Mass spectra were acquired in positive-ion mode using APCI with selected ion monitoring (SIM). The mass range was recorded from 100 to 1200 *m*/*z*. The obtained mass spectral profile was used to characterize the chemical fingerprint of the HF33 ethanolic extract.

### 4.5. Determination of Total Phenolic Content, Total Flavonoid Content, and Antioxidant Activity of HF33 Ethanolic Extract

#### 4.5.1. Total Phenolic Content

The total phenolic content (TPC) of the HF33 ethanolic extract was quantified using a Folin–Ciocalteu colorimetric assay. Briefly, the extract was combined with diluted Folin–Ciocalteu reagent and sodium carbonate solution. The resulting mixture was then incubated in the dark at room temperature, after which its absorbance was recorded at 630 nm using a microplate reader (BioTek, Winooski, VT, USA). A calibration curve prepared with gallic acid was employed for quantification, and the TPC was expressed as milligrams of gallic acid equivalents per gram of extract (mg GAE/g extract). All measurements were performed in triplicate.

#### 4.5.2. Total Flavonoid Content

The total flavonoid content (TFC) was assessed using an aluminum chloride colorimetric assay. The HF33 ethanolic extract was reacted with aluminum chloride and potassium acetate, followed by incubation at room temperature for 30 min. The absorbance of the resulting reaction mixture was measured at 415 nm using a microplate reader. Quantification was based on a quercetin calibration curve prepared over the concentration range of 10–500 μg/mL. The TFC was reported as milligrams of quercetin equivalents per gram of extract (mg QE/g extract). Each measurement was performed in triplicate.

#### 4.5.3. DPPH Radical Scavenging Assay

The radical-scavenging activity of the HF33 ethanolic extract was evaluated based on its ability to reduce DPPH absorbance. Various concentrations of the extract were prepared, and 20 μL of each sample was mixed with 180 μL of DPPH solution (80 μg/mL in methanol) in a 96-well microplate (BioTek, Winooski, VT, USA). The reaction mixtures were incubated in the dark at room temperature for 30 min. Subsequently, absorbance was determined at 517 nm. Gallic acid was included as the reference standard, whereas methanol was used as the control. The radical-scavenging activity was calculated relative to the control, and the half-maximal inhibitory concentration (IC_50_) was determined from the concentration–response relationship by linear regression analysis. All measurements were performed in triplicate.

#### 4.5.4. ABTS Radical Scavenging Assay

ABTS radical-scavenging activity was determined using an ABTS radical cation generated by reacting ABTS with potassium persulfate. The resulting mixture was incubated in the dark for 16 h and subsequently diluted with methanol to obtain an absorbance of 0.700 ± 0.02 at 630 nm before use. For the assay, 10 μL of each HF33 ethanolic extract sample was mixed with 190 μL of the ABTS working solution and incubated at room temperature for 15 min. The absorbance was then measured at 630 nm. Trolox (Sigma-Aldrich, St. Louise, USA) was used as the reference standard, and the IC_50_ value was calculated from the concentration–response relationship using linear regression analysis. All measurements were performed in triplicate.

#### 4.5.5. Ferric Reducing Antioxidant Power (FRAP) Assay

Ferric reducing antioxidant power of the HF33 ethanolic extract was assessed based on the reduction of the Fe^3+^–TPTZ complex to its Fe^2+^ form. The FRAP working reagent was freshly prepared from acetate buffer, TPTZ, and ferric chloride solutions at a ratio of 10:1:1. Extract samples were reacted with the working reagent and incubated at room temperature before absorbance measurement at 595 nm. Ferrous sulfate (FeSO_4_) was used to construct the calibration curve. FRAP activity was calculated from the calibration curve and expressed as mM Fe(II)/g extract. All measurements were performed in triplicate.

### 4.6. Experimental Animals and Ethical Statement

Sprague–Dawley rats were purchased from Nomura Siam International Co., Ltd. (Bangkok, Thailand) and housed in pairs under controlled environmental conditions (21 ± 2 °C, 12 h light/dark cycle) with unrestricted access to standard laboratory chow and water. Before each experiment, the animals underwent a minimum 7-day acclimatization period in the experimental facility. Animals were considered eligible for inclusion if they exhibited normal general appearance, behaviour, feeding, respiration, and excretion, together with appropriate weight gain during acclimatization, according to predefined eligibility criteria established a priori. No animals or data points were excluded from any experimental group during the analysis.

A total of 138 male Sprague–Dawley rats (6–8 weeks old, body weight 200–250 g) were used for the gastroprotective studies, whereas 10 female Sprague–Dawley rats (6 weeks old, body weight 170–180 g) were used for the acute oral toxicity study in accordance with OECD Guideline 420. For the gastroprotective experiments, sample size was determined using G*Power software (version 3.9.1.7; Heinrich-Heine-Universität Düsseldorf, Düsseldorf, Germany), resulting in six animals per group.

For each experiment, animals were first categorized into body-weight ranges, and identification numbers were randomly assigned by drawing lots within each weight category to allocate animals to the experimental groups. This randomisation procedure resulted in comparable mean body weights among the groups. During treatment administration, animals from different experimental groups were treated in an alternating order rather than completing one group before proceeding to the next, to minimise potential confounding related to treatment order. For the stress-induced gastric ulcer model, animals from all experimental groups were included in each exposure round to minimise potential confounding related to the timing of experimental procedures.

Prior to conducting any procedures involving animals, ethical clearance for the study was secured from the Institutional Animal Care and Use Committee of the Faculty of Medicine, Chiang Mai University, Thailand (Approval No. 19/2566; 20 April 2023). The experimental procedures followed the principles outlined in the National Institutes of Health Guide for the Care and Use of Laboratory Animals.

Animal welfare was carefully considered throughout the experimental period, with appropriate precautions taken to limit pain and distress. General appearance, behaviour, food and water intake, the Rat Grimace Scale, signs of toxicity, and body weight were assessed according to predefined welfare criteria. A body-weight loss of ≥20% was considered a critical humane endpoint. Animals in the gastroprotective experiments were monitored throughout the experimental procedures, whereas those in the acute toxicity study were closely monitored for the first 6 h after dosing and then daily for 14 days. No unexpected adverse events or treatment-related clinical abnormalities were observed.

### 4.7. Acute Oral Toxicity Study

To evaluate the safety profile and potential systemic toxicity of the HF33 ethanolic extract, an acute oral toxicity study was conducted according to OECD Test Guideline 420 [19].

Ten female Sprague–Dawley rats (6 weeks old) were randomly allocated to control and HF33-treated groups (*n* = 5/group). The animals received a single oral dose of the HF33 ethanolic extract (2000 mg/kg) or the vehicle (5% Tween 80) by oral gavage. All animals were monitored once daily for 14 days for mortality, signs of toxicity, pain, or distress. Body weight was recorded before treatment and on days 7 and 14 after treatment. At the end of the observation period, all animals were euthanized and blood samples were collected for hematological and serum biochemical analyses. Coded blood samples were submitted to the Small Animal Hospital, Faculty of Veterinary Medicine, Chiang Mai University, for hematological and serum biochemical analyses without disclosure of treatment allocation. Major organs were collected, weighed, and fixed in 10% neutral-buffered formalin before submission to the Department of Pathology, Faculty of Medicine, Chiang Mai University, for histopathological processing and evaluation. Tissue samples were coded prior to submission, and histopathological processing and evaluation were performed without knowledge of treatment allocation. Relative organ weights were expressed as the corresponding organ weight normalized to 100 g of body weight.

### 4.8. Experimental Design for Gastric Ulcer Models

The gastroprotective effects of the HF33 ethanolic extract were evaluated in rats using three experimental models of gastric injury: restraint water immersion stress-induced gastric ulcer, indomethacin-induced gastric ulcer, and acidified ethanol (EtOH/HCl)-induced gastric ulcer. The three models were selected to represent distinct experimental mechanisms and clinically relevant factors associated with gastric mucosal injury, including stress, NSAID exposure, and alcohol-related injury. Male Sprague–Dawley rats were selected because this species is widely used in experimental studies of gastric mucosal injury and gastroprotection and provides established models for investigating the pathophysiological mechanisms underlying gastric ulcer formation. Before each experiment, rats were fasted for 24 h with free access to water. Water was withdrawn 1 h before ulcer induction. The HF33 ethanolic extract was administered orally at doses of 150, 300, and 600 mg/kg, based on the dose range used in a previous study of a polyherbal extract [49] to evaluate the gastroprotective effects of HF33 across different dose levels. Cimetidine (100 mg/kg) was selected as the reference drug in models in which suppression of gastric acid secretion was a relevant pharmacological mechanism, whereas misoprostol (100 μg/kg) was used in models emphasizing enhancement of mucosal defense and cytoprotection [49,60]. The normal and ulcer control groups received the vehicle (5% Tween 80) by oral gavage.

After ulcer induction, animals were euthanized by intraperitoneal injection of thiopental sodium (100 mg/kg). The stomachs were removed, opened along the greater curvature, rinsed with normal saline solution, and examined for gastric lesions. The ulcer index was determined a Carl Zeiss Stemi stereomicroscope (Carl Zeiss, Göttingen, Germany) equipped with a 10× eyepiece by measuring the total length of gastric lesions in each stomach. The mean ulcer index was calculated for each experimental group.

#### 4.8.1. Restraint Water Immersion Stress-Induced Gastric Ulcer Model

A total of 30 male Sprague–Dawley rats were randomly divided into five groups (*n* = 6/group): vehicle control group, cimetidine-treated reference group (100 mg/kg), and HF33 ethanolic extract-treated groups (150, 300, and 600 mg/kg). The animals received the vehicle, cimetidine, or HF33 ethanolic extract orally 1 h before ulcer induction. Gastric injury was induced by placing the rats individually in stainless-steel restraint cages and immersing them in a water bath maintained at 21 ± 1 °C up to the xiphoid level for 5 h. After stress exposure, all rats were euthanized, and gastric tissues were collected for ulcer index determination as described above.

#### 4.8.2. Indomethacin-Induced Gastric Ulcer Model

A total of 30 male Sprague–Dawley rats were randomly divided into five groups (*n* = 6/group): vehicle control group, misoprostol-treated reference group (100 µg/kg), and HF33 ethanolic extract-treated groups (150, 300, and 600 mg/kg). The animals received the vehicle, misoprostol, or HF33 ethanolic extract orally 1 h before oral administration of indomethacin (100 mg/kg). Five hours later, all rats were euthanized and the stomachs were collected for determination of ulcer index.

#### 4.8.3. Acidified Ethanol (EtOH/HCl)-Induced Gastric Ulcer Model

A total of 30 male Sprague–Dawley rats were randomly divided into five groups (*n* = 6/group): ulcer control group, misoprostol-treated reference group (100 µg/kg), and HF33 ethanolic extract-treated groups (150, 300, and 600 mg/kg). One hour after oral administration of HF33 ethanolic extract, misoprostol, or the vehicle, gastric injury was induced by oral administration of EtOH/HCl solution consisting of absolute ethanol (60 mL), 12 N hydrochloric acid (1.7 mL), and distilled water (38.3 mL) at a volume of 1 mL/rat. One hour after ulcer induction, all rats were euthanized, and gastric tissues were collected for ulcer index determination, histopathological examination, and evaluation of oxidative stress markers.

### 4.9. Histopathological Evaluation of Gastric Tissues

Gastric tissues obtained from the EtOH/HCl-induced gastric ulcer model were fixed in 10% neutral-buffered formalin and submitted to the Department of Pathology, Faculty of Medicine, Chiang Mai University. The tissues were processed, embedded in paraffin, and sectioned at 5 μm. Sections were stained with hematoxylin and eosin (H&E) to evaluate general tissue morphology and with periodic acid–Schiff (PAS) to assess gastric mucus preservation. Gastric tissue samples were coded prior to submission, and histopathological processing and evaluation were performed without knowledge of treatment allocation. Histological changes were examined using a light microscope (Olympus BX53, Olympus Corporation, Tokyo, Japan) at 20× magnification. Representative images were captured for comparison among experimental groups.

### 4.10. Determination of Gastric Oxidative Stress Markers

Gastric oxidative stress was evaluated using gastric tissues obtained from the EtOH/HCl-induced gastric ulcer experiment. Malondialdehyde (MDA) levels were determined as an indicator of lipid peroxidation, while superoxide dismutase (SOD) activity was measured as an indicator of endogenous antioxidant defense. A separate group of normal, non-ulcerated rats (*n* = 6) was included as a reference for baseline MDA and SOD levels. Gastric tissues were homogenized in ice-cold buffer and centrifuged to obtain the supernatant. For MDA and SOD analyses, gastric tissue samples were coded and analysed without disclosure of the treatment allocation to the analyst. MDA levels and SOD activity were measured using a Lipid Peroxidation Assay Kit (Colorimetric) (cat. no. ab233471, Abcam Ltd., Cambridge, UK) and a Superoxide Dismutase (SOD) Assay Kit (cat. no. MAK528, Sigma-Aldrich, St. Louis, MO, USA), respectively, according to the manufacturers’ instructions. To determine total protein in gastric tissue homogenates, the Bradford protocol was executed using bovine serum albumin for standardization. Data normalization was performed against the quantified protein content, expressing MDA as nmol/mg protein and SOD as U/mg protein.

### 4.11. Evaluation of Gastric Secretion Using the Pylorus Ligation Model

The effect of the HF33 ethanolic extract on gastric secretion was evaluated using the pylorus ligation model. A total of 18 male Sprague–Dawley rats were randomly divided into three groups (*n* = 6/group): vehicle control group, cimetidine-treated reference group (100 mg/kg), and HF33 ethanolic extract-treated group (600 mg/kg). One hour after oral administration of the HF33 ethanolic extract, cimetidine, or the vehicle, pylorus ligation was performed under thiopental sodium anesthesia (50 mg/kg, intraperitoneally). Five hours after ligation, the rats were euthanized by intraperitoneal injection of thiopental sodium (100 mg/kg). Gastric contents were collected, and gastric volume, pH, and total acidity were measured. Total acidity was determined by titration with 0.01 N NaOH and expressed as mEq/L.

### 4.12. Determination of Gastric Wall Mucus Content by the Alcian Blue Binding Assay

The effect of the HF33 ethanolic extract on gastric wall mucus content was evaluated in a separate acidified ethanol (EtOH/HCl)-induced gastric ulcer experiment using the alcian blue binding assay. A total of 24 male Sprague–Dawley rats were randomly divided into four groups (*n* = 6/group): normal group, ulcer control group, misoprostol-treated group, and HF33 ethanolic extract-treated group (600 mg/kg). Except for the normal group, gastric injury was induced by oral administration of EtOH/HCl following pretreatment with the vehicle, misoprostol, or the HF33 ethanolic extract. One hour after ulcer induction, all rats were euthanized and the stomach tissues were collected and weighed. Gastric tissues were immersed in 0.1% alcian blue solution for 2 h to allow binding of mucus-associated dye. The dye–mucus complex was extracted using 0.5 M magnesium chloride solution. Absorbance was measured at 580 nm using a microplate reader (Metertech, Taipei, Taiwan). Gastric wall mucus content was calculated and expressed as μg alcian blue/g stomach weight.

### 4.13. Statistical Analysis

Quantitative results are expressed as mean ± standard deviation (SD). Physicochemical characteristics, phytochemical measurements, and antioxidant assay data were summarized descriptively using mean ± SD and were not subjected to inferential statistical testing. Analyses of the in vivo experimental data were conducted with SPSS Statistics version 25.0 (SPSS Inc., Chicago, IL, USA). Prior to selecting the appropriate statistical test, the distribution of the data and equality of variances were evaluated using the Shapiro–Wilk and Levene’s tests, respectively.

For the gastroprotective experiments, data satisfying the assumptions of normality and equal variance were analyzed by one-way analysis of variance (ANOVA), with Dunnett’s post hoc test subsequently applied to compare the treatment groups with the control group. When the data were normally distributed but exhibited unequal variances, Welch’s ANOVA was performed, followed by the Games–Howell post hoc test. For data that did not meet the assumption of normality, the Kruskal–Wallis test was applied, followed by appropriate pairwise comparisons.

For the acute toxicity experiment, differences between the control and extract-treated groups were evaluated using an independent-samples *t*-test when the data were normally distributed with equal variances. Welch’s *t*-test was used when normality was satisfied but variances were unequal, whereas the Mann–Whitney U test was applied to non-normally distributed data. A *p*-value < 0.05 was considered indicative of statistical significance.

To assess the dose–response relationship of the HF33 ethanolic extract, linear regression analysis was performed using the administered dose (0, 150, 300, or 600 mg/kg) as the independent variable. The linear association was evaluated based on the regression coefficient, standardized regression coefficient (β), coefficient of determination (R^2^), and *p*-value.

## 5. Conclusions

The present study demonstrated that the ethanolic extract of HF33 exhibited gastroprotective activity in multiple experimental gastric ulcer models. HF33 significantly reduced gastric injury induced by restraint water immersion stress, indomethacin, and acidified ethanol. These protective effects were associated with preservation of gastric mucosal integrity, enhancement of the gastric mucus barrier, and attenuation of oxidative damage, without significant alteration of gastric secretion parameters under the conditions tested. Quality characterization of the formulation and chemical fingerprinting of the extract provided information supporting the identity, quality, and reproducibility of the tested material. In addition, no evidence of acute oral toxicity was observed following a single oral administration of 2000 mg/kg.

Therefore, the present findings provide preclinical evidence supporting further investigation of HF33 as a polyherbal formulation with potential gastroprotective properties. Further studies are required to identify the active constituents, elucidate the underlying molecular mechanisms, evaluate repeated-dose toxicity and long-term safety, and determine the translational relevance of these findings.

## Figures and Tables

**Table 1:** Major ions detected in the APCI mass spectrum of HF33 ethanolic extract (positive-ion mode, *m*/*z* 100–1200).

Molecular Mass in Positive Ion [M^+^H]^+^	Molecular Mass [M]	Intensity
121.689	120.689	3.9 × 10^7^
134.098	133.098	1.2 × 10^7^
187.552	186.552	1.2 × 10^7^
119.821	118.821	1.2 × 10^7^
122.672	121.672	7.3 × 10^6^
321.125	320.125	7.2 × 10^6^
135.970	134.970	6.6 × 10^6^
188.039	187.039	5 × 10^6^
146.391	145.391	5 × 10^6^
191.149	190.149	5 × 10^6^

**Table 2:** Total phenolic content, total flavonoid content, and in vitro antioxidant activity of HF33 ethanolic extract.

Test Compounds	DPPH IC_50_ (μg/mL)	ABTS IC_50_ (μg/mL)	FRAP value (mM Fe (II)/g Extract)	TPC (mg GAE/g Extract)	TFC (mg QE/g Extract)
HF33	26.40 ± 0.87	5.60 ± 1.34	4729.00 ± 205.20	584.90 ± 89.31	25.83 ± 0.03
Gallic acid	2.20 ± 0.21	NT	NT	NT	NT
Trolox	NT	3.70 ± 0.24	NT	NT	NT

Data are presented as mean ± SD from three independent experiments (*n* = 3). IC_50_, half-maximal inhibitory concentration; GAE, gallic acid equivalents, QE, quercetin equivalents; TPC, Total phenolic content; TFC, Total flavonoid content. Gallic acid and Trolox were used as reference standards for the DPPH and ABTS assays, respectively, whereas ferrous sulfate (FeSO_4_) was used to construct the calibration curve for the FRAP assay. NT, not tested.

**Table 3:** Hematological parameters following acute oral administration of HF33 ethanolic extract in female rats.

Parameters	Control	HF33 (2000 mg/kg)
Hematocrit (%)	41.66 ± 1.45	42.12 ± 0.94
Hemoglobin (g/dL)	13.66 *±* 0.38	14.64 ± 0.38 **
RBC (×10^12^/L)	7.38 ± 0.23	7.92 ± 0.29 *
MCV (fl)	56.48 ± 0.80	53.22 ± 1.28 **
MCH (pg)	18.52 ± 0.22	18.50 ± 0.45
MCHC (g/dL)	32.80 ± 0.70	34.78 ± 0.49 **
WBC (×10^9^/L)	5.03 ± 1.91	5.41 ± 0.71
Neutrophil (%)	12.51 ± 0.78	12.22 ± 2.33
Lymphocyte (%)	81.01 ± 4.77	79.10 ± 3.66
Monocyte (%)	5.01 ± 3.29	7.30 ± 2.32
Eosinophil (%)	1.47 ± 0.86	1.38 ± 0.64
Basophil (%)	0.00 ± 0.00	0.00 ± 0.00
Platelets (×10^9^/L)	8.67 ± 1.15	9.71 ± 0.49

Data are presented as mean ± SD (*n* = 5). * *p* < 0.05 and ** *p* < 0.01 versus control group. RBC, red blood cell count; MCV, mean corpuscular volume; MCH, mean corpuscular hemoglobin; MCHC, mean corpuscular hemoglobin concentration; WBC, white blood cell count.

**Table 4:** Serum biochemical parameters following acute oral administration of HF33 ethanolic extract in female rats.

Parameters	Control	HF33 (2000 mg/kg)
Glucose (mg/dL)	162.60 ± 10.41	156.60 ± 18.84
BUN (mg/dL)	24.66 ± 6.54	25.48 ± 5.62
Creatinine (mg/dL)	0.67 ± 0.15	0.59 ± 0.10
AST (U/L)	95.20 ± 16.08	87.60 ± 16.46
ALT (U/L)	31.00 ± 7.38	27.60 ± 1.82
ALP (U/L)	54.40 ± 12.22	64.20 ± 11.17
Total bilirubin (mg/dL)	0.10 ± 0.03	0.10 ± 0.03
Direct bilirubin (mg/dL)	0.06 ± 0.02	0.07 ± 0.01
Total protein (g/dL)	6.24 ± 0.63	5.92 ± 0.43
Albumin (g/dL)	3.86 ± 0.55	3.82 ± 0.25
Globulin (g/dL)	2.38 ± 0.93	2.10 ± 0.19

Data are presented as mean ± SD (*n* = 5). No significant differences were observed between groups. BUN, blood urea nitrogen; AST, aspartate aminotransferase; ALT, alanine aminotransferase; ALP, alkaline phosphatase.

**Table 5:** Relative organ weights following acute oral administration of HF33 ethanolic extract in female rats.

Organ	Control	HF33 (2000 mg/kg)
Brain	0.96 ± 0.02	0.95 ± 0.03
Lung	0.67 ± 0.05	0.67 ± 0.05
Heart	0.42 ± 0.03	0.39 ± 0.02
Liver	4.13 ± 0.27	4.02 ± 0.15
Spleen	0.26 ± 0.02	0.24 ± 0.03
Thymus	0.26 ± 0.03	0.27 ± 0.03
Adrenal gland	0.02 ± 0.00	0.02 ± 0.01
Kidney	0.02 ± 0.00	0.02 ± 0.00
Ovary	0.04 ± 0.01	0.03 ± 0.01
Uterus	0.51± 0.03	0.52 ± 0.04

Data are presented as mean ± SD (*n* = 5). No significant differences were observed between groups.

## Data Availability

The original contributions presented in this study are included in the article/Supplementary Material. Further inquiries can be directed to the corresponding authors.

## Supplementary Materials

The following supporting information can be downloaded at: https://www.mdpi.com/article/10.3390/ph19101565/s1, Figure S1: Representative microscopic characteristics of HF33 formulation powder; Table S1: Physicochemical quality parameters of HF33 formulation powder.

## Abbreviations

The following abbreviations are used in this manuscript:

ABTS	2,2′-azinobis(3-ethylbenzothiazoline-6-sulfonic acid)
ALP	Alkaline phosphatase
ALT	Alanine aminotransferase
APCI	Atmospheric-pressure chemical ionization
ANOVA	Analysis of variance
AST	Aspartate aminotransferase
BSA	Bovine serum albumin
BUN	Blood urea nitrogen
CMS	Compact mass spectrometry
DHE	Dihydroethidium
DPPH	2,2-diphenyl-1-picrylhydrazyl
EtOH/HCl	Acidified ethanol
FRAP	Ferric reducing antioxidant power
GAE	Gallic acid equivalents
H&E	Hematoxylin and eosin
HF33	Polyherbal formulation No. 33
HPLC-PDA	High-performance liquid chromatography with photodiode array detection
IC_50_	Half maximal inhibitory concentration
LD_50_	Median lethal dose
MCH	Mean corpuscular hemoglobin
MCHC	Mean corpuscular hemoglobin concentration
MCV	Mean corpuscular volume
MDA	Malondialdehyde
NSAID	Non-steroidal anti-inflammatory drug
OECD	Organisation for Economic Co-operation and Development
PAS	Periodic acid–Schiff
QE	Quercetin equivalents
RBC	Red blood cell count
SIM	Selected ion monitoring
SOD	Superoxide dismutase
TFC	Total flavonoid content
TPC	Total phenolic content
TPTZ	2,4,6-tris(2-pyridyl)-s-triazine
WBC	White blood cell count
