# Supplementary material for: Gastroprotective Effects of a Polyherbal Formulation (HF33) in Experimental Gastric Ulcer Models and Its Acute Oral Toxicity Evaluation
Source: Pharmaceuticals (Basel). 2026 Oct 1;19(10):1565. doi: 10.3390/ph19101565 (PMC13708707; doi:10.3390/ph19101565)
Supplement: Supplementary file 1 [file pharmaceuticals-19-01565-s001.zip › Supplementary Materials_Table S1.pdf]

# Gastroprotective Effects of a Polyherbal Formulation (HF33) in Experimental Gastric Ulcer Models and Its Acute Oral Toxicity Evaluation

Natthakarn Chiranthanut [a,*], Sunee Chansakaow [b], Phraepakaporn Kunnaja [c], Nirush Lertprasertsuke [d], Ratchadawan Puangpradab [e], and Nut Koonrungsesomboon [a,*]

**Table S1.** Physicochemical quality parameters of HF33 formulation powder.

| Specification | Content (%) by dried weight |
|---|---|
| Loss on drying | 7.67 ± 0.06 |
| Total ash | 5.34 ± 0.05 |
| Acid-insoluble ash | 1.62 ± 0.04 |
| Ethanol-soluble extractive value | 25.43 ± 0.07 |
| Water–soluble extractive value | 27.38 ± 0.06 |

Data are presented as mean ± SD from three independent experiments (n = 3).
