# Supplementary material for: Gastroprotective Effects of a Polyherbal Formulation (HF33) in Experimental Gastric Ulcer Models and Its Acute Oral Toxicity Evaluation
Source: Pharmaceuticals (Basel). 2026 Oct 1;19(10):1565. doi: 10.3390/ph19101565 (PMC13708707; doi:10.3390/ph19101565)
Supplement: Supplementary file 1 [file pharmaceuticals-19-01565-s001.zip › Supplementary Materials_Figure S1.pdf]

Gastroprotective Effects of a Polyherbal Formulation (HF33) in Experimental Gastric Ulcer Models and Its Acute Oral Toxicity Evaluation

Natthakarn Chiranthanut [a,*], Sunee Chansakaow [b], Phraepakaporn Kunnaja [c], Nirush Lertprasertsuke [d], Ratchadawan Puangpradab [e], and Nut Koonrungsesomboon [a,*]

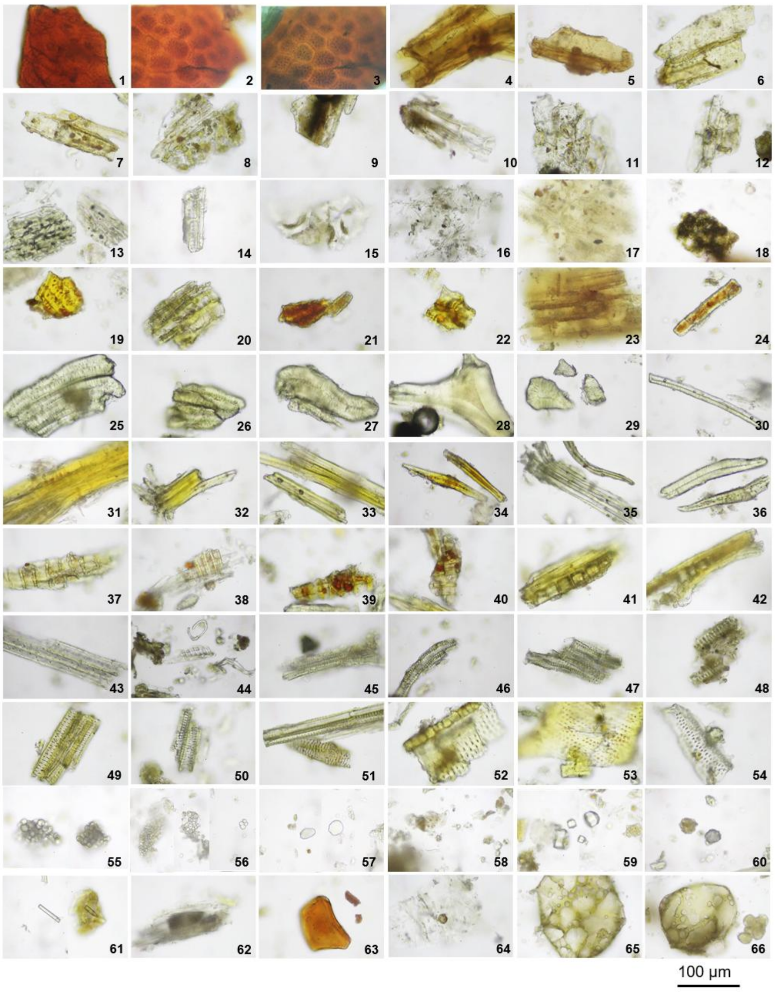

Figure S1. Representative microscopic characteristics of HF33 formulation powder.

Diagnostic microscopic features observed in the powdered formulation include: (1-6) cork cells; (7-10) epidermis; (11) epidermis with stomata; (12) epidermis with cicatrix; (13-17) parenchyma containing various inclusions; (18) chlorenchyma; (19-24) thick-walled parenchyma containing reddish-brown sub-stances; (25-30) sclereids; (31-36) fibers; (37-40) medullary rays; (41-42) prism sheath and fibers; (43) fiber-tracheid; (44-48) spiral vessels; (49-51) pitted vessels; (52-54) bordered pitted vessels; (55-57) starch grains; (58) oil droplet;

(59) prismatic crystals; (60) rosette crystals; (61) rod crystals; (62) raphide crystals; (63) brown matter; (64) aleurone grain; and (65-66) yellowish masses. Original magnification, ×40; Scale bar = 100 μm.
